# Heard Island glacier albedo from VIIRS satellite observations 2012 to 2024

**DOI:** 10.1038/s41597-026-07783-2

**Published:** 2026-07-30

**Authors:** Wang Lin, Wang Feiteng, Du Zhencai, Ming Jing

**Affiliations:** 1 Key Laboratory of Cryospheric Science and Frozen Soil Engineering, Northwest Institute of Eco-Environment and Resources, Chinese Academy of Sciences, Lanzhou, 730000 China; 2 National Field Observation and Research Station (Xinjiang) for Tianshan Glaciers, Northwest Institute of Eco-Environment and Resources, Chinese Academy of Sciences, Lanzhou, 730000 China; 3 Institute of Atmospheric Physics, Chinese Academy of Sciences, Beijing, 100029 China; 4 Beacon Science & Consulting, Tranmere, SA 5073 Australia

## Abstract

Glacier albedo controls surface energy balance and mass balance but remains poorly observed in remote sub-Antarctic regions. We present a glacier albedo dataset for Heard Island (53°06’S, 73°31’E) derived from NASA VIIRS VNP43 BRDF/albedo parameters for January 2012 to May 2024. The dataset includes a glacier-wide area-mean time series (4,466 daily albedo records) and annual albedo rasters at 375–500 m resolution. White-sky albedo (WSA) was computed from BRDF parameters with quality filtering (mandatory quality ≤ 1). Processing used Google Earth Engine with glacier masking and interior buffering (1 pixel, 375 m). Temporal data availability is 98.9%; spatial coverage is 44.04% of the glacier area due to cloud cover. The dataset is suitable for glacier surface energy balance studies, trend analysis, and integration with climate reanalysis. Data are provided in CSV and GeoTIFF formats with accompanying quality metadata.

## Background & Summary

**Fig. 1:**
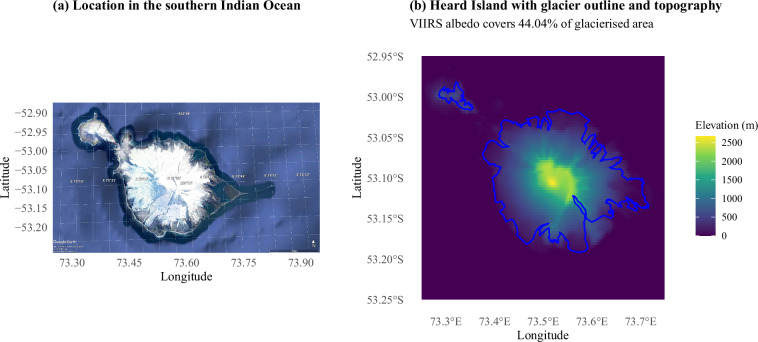
Study area and data coverage. (**a**) Location of Heard Island (53°06’S, 73°31’E) in the southern Indian Ocean. (**b**) Heard Island with glacier outline (blue, 226 km² as of 2019) and SRTM DEM-derived topography (elevation in m). The VIIRS albedo dataset covers 44.04% of the glacierised area. Data period: 2012–2024.

Glacier albedo—the fraction of solar radiation reflected by glacier surfaces—is a critical parameter for surface energy balance and mass balance modelling^1,2^. Sub-Antarctic islands host glacier systems that are sensitive to climate change but remain among the least studied components of the global cryosphere due to extreme remoteness^3,4^. Heard Island (53°06’S, 73°31’E) is a remote volcanic island in the southern Indian Ocean, approximately 4,000 km southwest of Perth, Australia. The island covers 368 km², rises to 2,745 m at Mawson Peak, and is dominated by Big Ben, an active volcano (Fig. 1). Glaciers occur primarily on eastern and southern flanks, with major outlet systems including the Gotley, Compton, and Stephenson Glacier complexes^5^. Glacier area declined from 289 km² in 1947 to 226 km² in 2019^6^, yet long-term albedo datasets for sub-Antarctic glaciers have been lacking.

The NASA VIIRS (Visible Infrared Imaging Radiometer Suite) instrument provides BRDF/albedo parameters from the VNP43 product, enabling albedo retrievals with angular corrections^7,8^. This dataset addresses the gap by providing the first comprehensive glacier albedo archive for Heard Island, derived from VIIRS observations. The data are suitable for surface energy balance studies, temporal trend analysis, integration with climate reanalysis, and comparison with other cryospheric datasets. Potential reuse includes glacier mass balance modelling, climate-glacier relationship studies, and validation of regional climate models.

## Methods

### Input data

VIIRS BRDF/Albedo: We used the NOAA VIIRS VNP43 product (VNP43IA1 for BRDF parameters, VNP43IA2 for quality flags) extracted on 20 October 2025 from Google Earth Engine (GEE)^9^ public data catalogue (collection ID: NOAA/VIIRS/001/VNP43IA1 and NOAA/VIIRS/001/VNP43IA2). The VNP43 product provides 16-day composite BRDF/albedo model parameters at 500 m resolution (native VIIRS resolution varies 375–500 m depending on scan angle). However, processing and export in GEE were performed at 375 m pixel scale to match the finer VIIRS I-band resolution, which is the native scale at which the VNP43 product is also available. Data were filtered for the Heard Island glacier extent from January 18, 2012 to May 31, 2024.

Glacier outline: Glacier boundaries were obtained from the Global Land Ice Measurements from Space (GLIMS) glacier database, compiled and made available by the international GLIMS community and the National Snow and Ice Data Center (NSIDC), Boulder CO, U.S.A. (10.7265/N5V98602, updated 2018))^10^. The outline was used for masking and spatial aggregation.

Digital elevation model: SRTM 1 arc-second DEM, accessed via GEE (collection ID: USGS/SRTMGL1_003), originally produced by NASA/USGS and publicly distributed through the USGS Earth Explorer platform (https://earthexplorer.usgs.gov/), was used for topographic context. A resampled version matching VIIRS resolution (375 m) is provided with the dataset.

ERA5-Land daily climate reanalysis: Accessed via GEE (collection ID: ECMWF/ERA5_LAND/DAILY_AGGR) and originally provided by the European Centre for Medium-Range Weather Forecasts (ECMWF) through the Copernicus Climate Data Store (CDS, https://cds.climate.copernicus.eu/).

### Albedo calculation

Broadband white-sky albedo (WSA) was derived from BRDF parameters using the formulation^7^:

$${\rm{WSA}}={{\rm{f}}}_{{\rm{iso}}}+0.189184\times {{\rm{f}}}_{{\rm{vol}}}-1.377622\times {{\rm{f}}}_{{\rm{geo}}}$$

where f_iso_, f_vol_, and f_geo_ represent the isotropic, volumetric, and geometric scattering components of the BRDF model. WSA, bihemispherical reflectance under isotropic illumination was selected because it is the standard for snow and ice surfaces^11^ and is independent of solar geometry, facilitating temporal comparison. For direct-beam dominated (clear-sky) conditions, black-sky albedo (BSA) would in principle better represent the instantaneous surface energy balance at a given solar zenith angle. However, WSA represents the diffuse limit and is the standard quantity used in snow and ice surface energy balance literature. Given the frequently overcast conditions at Heard Island, the diffuse assumption is often a reasonable approximation of real illumination conditions. Users requiring BSA for clear-sky energy balance calculations may compute it from the BRDF parameters provided in the VNP43 product.

### Quality control

We applied conservative quality filtering using the BRDF_Albedo_Band_Mandatory_Quality flags (I1, I2, I3 bands). Only pixels with mandatory quality ≤ 1 were retained (0 = best/full inversion, 1 = good; values 2 = fair and 3 = magnitude inversion were excluded). This threshold minimises contamination from clouds, shadows, and poor-quality retrievals.

### Spatial processing

All processing was performed in Google Earth Engine. Images were projected to EPSG:4326 (WGS84) at 375 m scale. Glacier-area masking was applied using the inventory outline. Interior buffering was implemented using a 1-pixel (375 m) focal minimum to exclude edge pixels and minimise mixed-pixel effects at glacier boundaries^12^.

### Temporal aggregation

For the area-mean time series, daily area-mean albedo record based on every 16-day measurements was computed from all valid pixels within the buffered glacier mask for each observation date. For annual rasters, 16-day composites were aggregated to yearly mosaics using the mean of valid observations per pixel per calendar year.

### Software and reproducibility

Processing used Google Earth Engine (JavaScript) and R (version 4.5.2), tidyverse [2.0.0], terra [1.8–42], and sf [1.0–21]. The GEE VIIRS collection versions are NOAA/VIIRS/001/VNP43IA1 and NOAA/VIIRS/001/VNP43IA2 (Version 001). Scripts are available in the accompanying repository (see Code Availability).

## Data Records

The dataset described here has been deposited in the Zenodo repository and is openly available under a Creative Commons Attribution 4.0 International (CC-BY 4.0) licence at 10.5281/zenodo.20821897 (ref.^13^).

### Primary dataset: VIIRS glacier albedo

**File 1: heard_island_viirs_albedo_area_mean.csv**

Description: Glacier-wide area-mean albedo time series

Format: CSV (comma-separated, UTF-8)

Columns: date (YYYY-MM-DD), albedo (unitless, 0–1)

Temporal coverage: January 18, 2012 to May 31, 2024

Number of records: 4,466

Temporal resolution: One value per observation date with valid data

**File 2: heard_island_viirs_quality_area_mean.csv**

Description: Quality flag time series for the area-mean albedo

Format: CSV

Columns: date, quality_i1, quality_i2, quality_i3, valid_days_i1, valid_days_i2, valid_days_i3, snow_flag, land_water_type, product, source (See the below table)

Purpose: Supports quality assessment and filtering of the albedo series

**Table:** 

Column	Type	Description
date	YYYY-MM-DD	Observation date
quality_i1	Integer (0–3)	Mean mandatory quality flag for band I1 (0 = best/full inversion, 1 = good, 2 = fair, 3 = magnitude inversion)
quality_i2	Integer (0–3)	Mean mandatory quality flag for band I2
quality_i3	Integer (0–3)	Mean mandatory quality flag for band I3
valid_days_i1	Integer	Number of valid input days contributing to the I1 composite
valid_days_i2	Integer	Number of valid input days contributing to the I2 composite
valid_days_i3	Integer	Number of valid input days contributing to the I3 composite
snow_flag	Integer	BRDF/albedo snow flag (0 = snow-free; 1 = snow)
land_water_type	Integer	Land/water type flag from the VNP43 product
product	String	Source product identifier (e.g., VNP43IA2)
source	String	Data collection source (e.g., GEE collection ID)

**File 3: heard_island_viirs_albedo_yearly_YYYY.tif (one file per year, 2012–2024)**

Description: Annual rasters are computed as the mean of all valid daily albedo values per pixel within each calendar year; pixels with no valid observations in a given year are assigned the NoData value (NaN)

Format: GeoTIFF

Spatial resolution: Data processed and exported at 375 m pixel scale from VIIRS VNP43 500 m BRDF parameters.

Projection: EPSG:4326 (WGS84)

Units: Albedo (unitless, 0–1)

NoData:NaN for masked/invalid pixels

**Supporting data**

**File 4: heard_island_era5_land_climate_daily.csv**

Description: Daily climate reanalysis for the study area

Source: ECMWF ERA5-Land^14^

Columns: date, temperature_2m (°C), precipitation (mm), snowfall (mm), surface_solar_radiation_downwards (W/m²)

Temporal resolution: Daily

Spatial resolution: ERA5-Land native ~9 km (area-mean for Heard Island)

**File 5: heard_island_srtm_dem_resampling_viirs.tif**

Description: SRTM DEM resampled to VIIRS resolution

Format: GeoTIFF

Units: Metres above sea level

Projection: EPSG:4326

**File 6: HIG.shp (and associated.dbf,.prj,.shx)**

Description: Glacier outline shapefile

Source: GLIMS and NSIDC (2005, updated 2018)^10^

## Data Overview

**Fig. 2:**
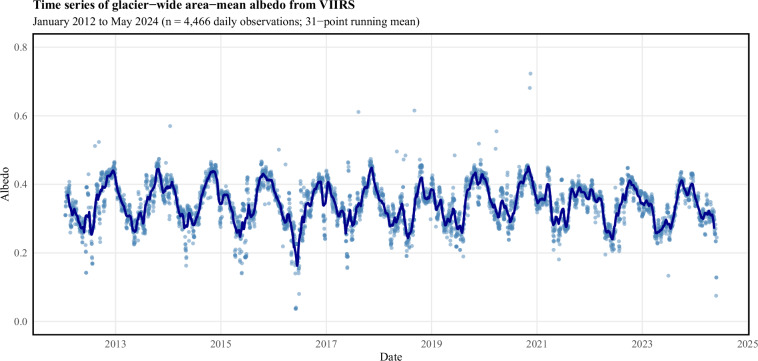
Time series of glacier-wide area-mean albedo from VIIRS (18 January, 2012 to 31 May, 2024). Points show daily values (n = 4,466); Thick blue line shows 31-point running mean. Albedo range 0.037–0.723 reflects seasonal and interannual variability in surface conditions.

The dataset comprises 4,466 area-mean albedo observations on the day-record basis spanning 2012–2024. Albedo values range from 0.037 to 0.723 (mean 0.343 ± 0.062 SD), encompassing conditions typical of bare ice, melting snow, and seasonal snow cover^15,16^. Temporal data availability is 98.9% of days in the study period; spatial coverage of valid pixels is 44.04% of the glacier area due to persistent cloud cover characteristic of sub-Antarctic maritime climates (Fig. 2).

## Technical Validation

### Algorithm validation

The VNP43 BRDF/albedo product has been validated against ground measurements and MODIS continuity^7,8^. The WSA formulation uses coefficients derived from radiative transfer modelling^11^.

### Internal consistency

We assessed temporal consistency by comparing albedo distributions across sub-periods (2012–2016, 2017–2020, 2021–2024). No evidence of sensor drift or systematic temporal artefacts was found. Albedo values fall within the expected range for snow and ice surfaces^15,16^.

### Spatial coverage

The 44.04% spatial coverage reflects cloud frequency and sensor geometry. Coverage is typical for polar and sub-polar regions where persistent cloud limits clear-sky observations^17^. The interior buffer (1 pixel) reduces mixed-pixel contamination at glacier edges. This represents the removal of one ring of edge pixels around the glacier perimeter, which is standard practice to reduce mixed-pixel effects at glacier margins.

### Quality flag distribution

The majority of retained observations have mandatory quality 0 (full inversion) or 1 (good). Excluded quality levels (2, 3) represent degraded retrievals under cloudy or low-observation conditions. At quality ≤ 1, temporal coverage is near-complete and annual pixel coverage exceeds 98%, whereas the lower mean daily spatial coverage (44.04%) mainly reflects cloud-limited clear-sky sampling; sensitivity to relaxed quality thresholds is reported in Technical Validation.

### Comparison with excluded sensors

MODIS provided 0% coverage over Heard Island in GEE for the study period. The 0% MODIS coverage reflects the fact that the Heard Island glacier extent (226 km²) falls near the boundary of MODIS sinusoidal grid tiles in GEE, and the available GEE MODIS collection (MODIS/061/MCD43A3) does not provide valid retrievals over this location — likely because the island’s small area and frequent cloud cover result in insufficient clear-sky observations to complete the 16-day BRDF inversion for this product version. This limitation motivated the use of the VIIRS VNP43 product, which achieves 98.9% temporal availability over the same period, demonstrating its superior performance for this location. Landsat and Sentinel-2/3 were evaluated; VIIRS was selected for superior temporal consistency (98.9% availability) and validated BRDF parameters. Sentinel-3 uses adapted coefficients from Sentinel-2 MSI rather than OLCI-specific coefficients^18^, introducing additional uncertainty.

### Estimate of uncertainty

(1) the spatial standard deviation of annual-composite albedo across valid glacier pixels, averaged over 2012–2024: mean SD = 0.140 (range 0.128–0.153), reflecting genuine within-glacier variability in surface conditions (bare ice vs. snow-covered areas) rather than retrieval noise alone; (2) the temporal variability of the area-mean time series (SD = 0.062 as reported in the Abstract) as context for interannual variability; and (3) reference to the global VNP43 product uncertainty characterisation, which reports typical absolute retrieval accuracy of ± 0.03–0.05 for snow and ice surfaces.

### Limitations

(1) the 44.04% spatial coverage reflects persistent cloud cover typical of the sub-Antarctic maritime climate, meaning clear-sky albedo values may not be fully representative of all-sky or cloudy conditions; (2) the glacier outline is a static boundary from GLIMS (2005, updated 2018), and does not capture glacier retreat over the 2012–2024 study period — users undertaking pixel-level analysis of retreating termini should apply dynamic outlines; (3) each daily observation is derived from a 16-day compositing window.

## Usage Notes

### Temporal matching

The area-mean time series provides one value per observation date, but consecutive values are not temporally independent because the VNP43 BRDF inversion uses a 16-day sliding compositing window. Users performing trend analysis or autocorrelation-sensitive statistics should account for this temporal structure.

### Spatial resolution mismatch

When combining with ERA5-Land climate data, note the spatial scale difference: albedo is at 375 m; ERA5-Land is ~9 km. Area-mean albedo is appropriate for glacier-wide comparisons. Pixel-level analysis should consider elevation-dependent climate gradients^19^.

### Glacier outline

The glacier outline is from the GLIMS database (2005, updated 2018)^10^. Glacier boundaries have changed over the study period; users may wish to apply dynamic outlines for pixel-level analysis of retreating termini.

### Reproducing the dataset

The GEE script (viirs.js) can be run to reproduce exports. Users need a GEE account and access to the Heard Island glacier asset. Parameter values (date range, quality threshold, buffer) are specified in the script.

## Data Availability

The Heard Island VIIRS glacier albedo dataset (2012–2024) is available at https://github.com/petermingjing/heard_island. The dataset includes: heard_island_viirs_albedo_area_mean.csv, heard_island_viirs_quality_area_mean.csv, annual albedo GeoTIFFs (2012–2024), heard_island_era5_land_climate_daily.csv, heard_island_srtm_dem_resampling_viirs.tif, and the glacier outline shapefile. All files are released under the Creative Commons Attribution 4.0 International (CC-BY 4.0) licence.

## Code availability

All processing and analysis code is included in the same Zenodo deposit (10.5281/zenodo.20821897; ref. ^13^), in the code/ directory. The repository includes: (1) viirs.js – Google Earth Engine script for VIIRS albedo export; (2) climate.js – GEE script for ERA5-Land export; (3) srtm_export.js – GEE script for DEM export; (4) R scripts for descriptive statistics and visualisation. R version 4.0 or higher was used. Key packages: tidyverse, terra, sf. Instructions to reproduce the dataset and figures are provided in the repository README.
